# A review of platelet-rich plasma for enteric fistula management

**DOI:** 10.3389/fbioe.2023.1287890

**Published:** 2023-11-15

**Authors:** Shuang Liang, Zhiqiang Zheng, Yaxin Li, Yuanming Yang, Lifeng Qin, Zhen Zhao, Licun Wang, Haiyan Wang

**Affiliations:** ^1^ Department of Blood Transfusion, The Affiliated Hospital of Qingdao University, Qingdao, China; ^2^ Department of Clinical Laboratory, Zhangdian District People’s Hospital of Zibo City, Zibo, China; ^3^ Department of Emergency Surgery, The Affiliated Hospital of Qingdao University, Qingdao, China

**Keywords:** enteric fistula, platelet-rich plasma, treatment, growth factor, tissue healing

## Abstract

Enteric fistula (EF), a serious complication after abdominal surgery, refers to unnatural communication between the gastrointestinal tract and the skin or other hollow organs. It is associated with infection, massive fluid/electrolyte loss, and malnutrition, resulting in an unhealed course. Despite advances in surgical techniques, wound care, infection control, and nutritional support, EF remains associated with considerable morbidity and mortality. Autologous platelet-rich plasma (PRP) containing elevated platelet concentrations has been proposed to promote healing in many tissues. However, the mechanism of action of PRP in EF treatment remains unclear owing to its complicated clinical manifestations. In this review, we summarized the clinical approaches, outlined the principal cytokines involved in the healing effects, and discussed the advantages of PRP for EF therapy. In addition, we defined the mechanism of autologous PRP in EF management, which is essential for further developing EF therapies.

## 1 Introduction

Enteric fistulas (EF) are pathological channels between the gastrointestinal tract and other hollow organs, including the body cavities, or outside the body. This causes intestinal contents to flow out of the intestinal lumen into other organs or outside the body, firstly, due to the loss of digestive fluid, patients may suffer from obvious imbalance of water, electrolyte disturbances, disorder of internal environment, and insufficiency of the circulating blood volume. Secondly, as the lost nutrients cannot be replenished by the gastrointestinal tract, nutritional deficiencies in turn lead to metabolism disorders and immune dysregulation. Moreover due to the stress exposure, catabolism increases, which may lead to negative nitrogen balance and hypoproteinemia in patients. EF can occur spontaneously or as a result of intra-abdominal surgery. The incidence rate of spontaneous fistula is approximately 15%–25% (Berry and Fischer, 1996; Arenas-Marquez et al., 1999). These can be a secondary consequence of Breakdown of Crohn’s disease, malignancy, radiation exposure, infectious diseases, diverticular disease, vascular failure, and mesenteric ischemia (Metcalf, 1999). Postoperative fistulas account for 75%–85% of all fistulas in the gastrointestinal tract and result from inadvertent enterotomy, rupture at the site of anastomosis, a foreign object proximal to the anastomosis, poor suturing technique, distal obstruction, hematoma, abscess formation at the anastomotic site, or tumor (Berry and Fischer, 1994; Metcalf, 1999; Evenson and Fischer, 2006). The morbidity and mortality associated with postoperative fistulas are significant, as they are highly associated with nutritional deficiencies, septic complications, and comorbidities that can occur during prolonged hospital stays (Evenson and Fischer, 2006).

Patients with enterocutaneous fistulas may experience significant weight loss due to prolonged periods of impaired nutrient absorption and the loss of large amounts of nitrogenous material from the fistula. To date, infection remains the major cause of treatment failure in patients with parenteral fistulas (Martinez et al., 2008; Polk and Schwab, 2012). For extra-intestinal fistula, intestinal bacteria can induce severe abdominal infection, imbalance of homeostasis, septic shock, multiple organs dysfunction syndrome, resulting in difficulty in treatment, prolonged hospitalization, increased costs and mortality. Before 1960, the mortality rate of EF was approximately 60% owing to homeostatic and metabolic challenges caused by massive fluid/electrolyte loss and reduced nutrient resorption (Edmunds et al., 1960; Monod-Broca, 1977). With advancements in medical care, infection control, and adequate nutritional support, the mortality rate has decreased by 10%–30% (Martinez et al., 2008). Pharmacologic approaches based on systemic administration of antibiotics, somatostatin, and immunomodulators result in poor therapeutic outcomes (Berger et al., 2021). Brooks et al. (2021) examine the impact of intestinal fistulas on the U.S. healthcare system between 2004 and 2014. The average annual incidence of cases was 28,845, the median length of hospital stay was 8 days, and the median total cost of hospitalization was $16,250. Inpatient fistula care is associated with a total of 230,000 hospital days and 86,000 procedures annually, with the number of annual cases and costs increasing over time.

However, EF still causes great pain to patients because of its difficult treatment, long cycle, high cost, serious effects on their physical and mental health, reduced quality of life and work, and heavy nursing and economic burden to the family. Thus, it is considered one of the most difficult problems in surgery. The treatment of EF requires an individualized treatment plan from the healthcare team. Patients without life-threatening complications receive conservative management with conservative management of fistula output, fluid and electrolyte replacement, nutritional support, and antibiotic therapy. Patients with life-threatening complications require salvage surgery, which can lead to increased morbidity and mortality and prolonged hospitalization. Cellular therapy has recently been investigated as an alternative treatment due to the unmet need for post-surgical fistula treatment.

Platelet-rich plasma (PRP) is a blood-derived product that has gained increasing attention in recent years due to its potential therapeutic benefits in various medical applications. PRP is prepared by extracting a small amount of blood from the patient, and then processing it in a centrifuge to separate the platelets and growth factors from other blood components. The resulting PRP concentrate can then be injected or applied topically to the injured area. The current state of PRP research suggests that it can enhance tissue regeneration and accelerate the healing process in a wide range of medical conditions including osteoarthritis, tendinopathy, and ligament injuries. More importantly, it has also shown promising results in wound healing, such as EF treatment.

Despite these promising prospects, there are still challenges in our understanding and practical applications of PRP. One of the main gaps is that there is currently no consensus on the optimal concentration of platelets, the most effective preparation method, or the best route of administration. This makes it difficult to compare the results of different studies and to establish evidence-based protocols for clinical use. Another gap is the limited understanding of the mechanisms by which PRP promotes tissue regeneration and accelerates the healing process. While it is known that PRP contains a high concentration of growth factors, these factors stimulate tissue repair and regeneration are still not fully understood. Further research is needed to elucidate these mechanisms and to identify the most effective strategies for using PRP in clinical practice. In this review, we provide a comprehensive overview of the clinical approaches for using platelet-rich plasma (PRP) in the treatment of EF. The article outlines the principal cytokines involved in the healing effects of PRP and highlights the advantages of PRP therapy for EF. Furthermore, the review defines the underlying mechanism by which PRP can be effective in managing EF, which is crucial for further development of EF therapies.

## 2 EF classification and treatment

An enterocutaneous fistula, classified according to anatomy, pathology, or physiology, is an abnormal connection that develops between the intestinal tract or stomach and the skin (Berry and Fischer, 1996). Anatomical classifications describe the intestinal segment from which the fistula originates, for example, mucocutaneous and colocutaneous fistulas. The etiological classification is based on the underlying disease process, such as trauma, foreign body, Crohn’s disease, malignancy, and iatrogenic causes (surgery, percutaneous drainage). Physiological classification is based on the volume of the fistula output: low output (<200 mL/d), medium output (200–500 mL/d), and high output (>500 mL/d). When the intestinal cavity communicates with other hollow viscera, it is called an internal fistula, such as the gallbladder bile duct fistula or rectovaginal fistula. Internal fistulas lead to infection of the affected organs or inflammation due to erosion and irritation of the digestive tract fluid or contents, causing local erosion, ulcers, suppuration, and necrosis, developing into external fistulas.

Congenital fistulae are rare and mostly acquired. EF may occur in patients with malignancy, radiation exposure, or inflammatory disorders, such as inflammatory intestinal disease; however, it is more commonly a complication of gastrointestinal surgery (Kumpf et al., 2017). Urological intestinal fistulas commonly occur in patients with inflammatory intestinal disease, of which diverticulitis is the most common cause, accounting for approximately 65%–79% of cases. The second leading cause of fistulas is cancer (10%–20%), followed by Crohn’s disease (5%–7%) (Gill, 2016).

After EF diagnosis, an active treatment plan that considers the patient’s etiology, classification, and nutritional status should be formulated. Common treatments involve infection control, fistula management, nutritional support, occlusion therapy, and a suitable time for surgery (Metcalf, 2019).

### 2.1 Infection control

Controlling the infection source is a key step in EF treatment. Effective and rapid infection control can considerably improve the prognosis of patients with EF. Sepsis is a common condition and the main cause of death in patients with EF (Reber et al., 1978; Soeters et al., 1979; Rolandelli and Roslyn, 1996; Campos et al., 1999; Hollington et al., 2004; Bleier and Hedrick, 2010; Ashkenazi et al., 2017). Timely and effective treatment is required once sepsis is detected. The infection may be due to persistent anastomotic leakage, fistula effusion, or a treatment complication and should be treated with appropriate antibiotics guided by culture susceptibility. Intestinal fluids and abscesses in the abdominal cavity should be fully drained, washed, and treated using the double-cannula technique. Surgical sepsis control should focus on infection drainage and externalization of the source of infection in the small or large bowel, and anastomoses should not be performed in critically ill patients or in cases of severe purulent or fecal contamination. Resection of healthy bowel that may be involved in the inflammatory process should be avoided. Support of organ system function and utilization of intensive care unit care are often required (Gribovskaja-Rupp et al., 2016). Complete debridement of the fistula is required before platelet-rich plasma (PRP) treatment, and fistula infection should be controlled before treatment. Incomplete debridement or residual infected tissue affects the effectiveness of PRP treatment and leads to infection recurrence. Platelets also have antibacterial activity and play a role in innate immune defense by releasing various platelet antimicrobial proteins (Yeaman, 1997; Tang et al., 2002). *In vitro,* PRP has antibacterial effects on methicillin-resistant *Staphylococcus aureus*, *Escherichia coli*, Extended spectrum *beta-lactamase* (ESBL)-positive *Klebsiella pneumoniae,* and carbapenem-resistant *Pseudomonas aeruginosa* (Moojen et al., 2008; Cetinkaya et al., 2019). This antibacterial effect correlates with the number of leukocytes in the PRP. In addition, when the pH of the PRP is 6.5–6.7 (acidic environment) (Marx, 2004), it inhibits bacterial growth.

### 2.2 Conservative treatments

In addition to primary management of sepsis, conservative management remains the mainstay of care, including fistula management and fistula drainage control, nutritional support, and occlusion treatments.

#### 2.2.1 Treatment of fistula and fistula effluent control nutritional

The enzyme content in the fistula effluent, combined with the prolonged exposure of the perifistula skin to moisture, may cause the tissue surrounding the fistula to break down rapidly. This condition prevents spontaneous fistula closure and makes it susceptible to infection, causing distress to patients. Protecting the skin and surrounding soft tissues from contact with the fistula exudate is critical for successful conservative EF management. Fistula management is a top priority in the care of patients with enterocutaneous fistulas; it is critical to improving quality of life and requires a multidisciplinary team of nurses and physicians to develop a customized treatment plan for each patient and to continually assess the fistula and fistula drainage as it changes over time (Gribovskaja-Rupp et al., 2016). Effective skincare can protect the skin and surrounding soft tissues, promoting wound healing. To develop treatment plans, fistula effluent is collected to identify patients with nutritional loss after accurately measuring the output (Lloyd et al., 2006; Ashkenazi et al., 2017). In addition, PRP occlusion therapy is suitable for treating low-flow tubular fistulas.

#### 2.2.2 Nutritional support

High-volume enterocutaneous and internal fistulas that bypass the large intestinal segments can result in malnutrition. This may be related to the following factors: 1) reduced dietary nutrient intake, 2) increased protein requirements due to inflammation or infection, and 3) nutrient loss associated with fistula output. Malnutrition may impair wound healing and increase postoperative infection or complications (Windsor and Hill, 1988a; Windsor and Hill, 1988b; Meguid et al., 1988); therefore, nutritional support, including total parenteral and enteral nutrition, provides estimated nutrient requirements, maintains fluid and electrolyte balance, and enhances spontaneous closure of enterocutaneous fistulas when feasible. PN is necessary for most patients with high-output fistulas (Gribovskaja-Rupp et al., 2016). Especially in rare patients with high-output fistulas or bowel failure due to diffuse disease, PN may be the only option. However, PN is costly, some degree of deep vein thrombosis occurs in 40% of all peripherally placed vascular devices, and contraindications to PN include hepatic dysfunction/failure and difficulty with vascular access or infection of the vascular access device. Contraindications to EN include inadequate bowel length (<75 cm), bowel discontinuities, significant increase in fistula output at the start of EN resulting in electrolyte disturbances, intolerance of EN symptoms, and inability to establish/maintain nutritional access. (Gribovskaja-Rupp et al., 2016). Therefore, a complete nutritional assessment by a specialized nutrition support team and a treatment plan tailored to the individual patient based on pathway and volume is required.

#### 2.2.3 Occlusion treatments

Active closure therapy is an important measure in the clinical treatment to provide adequate drainage, skin protection, fluid/electrolyte balance, enteral or parenteral nutrition, wound care, antimicrobial therapy, and intestinal rest in patients with signs of systemic sepsis or painful inflammation. Occlusion of the fistula must be performed when tissues around the fistula are firm, no infection is present, and the digestive tract below the fistula is unobstructed. Occlusion treatment for fistulas is divided into two categories: external and internal occlusion. Commonly used external occlusion methods include patches, tubes, biological protein glue, and hydraulic methods. Fibrin glue containing high concentrations of fibrinogen (Fg) and thrombin is a type of biological protein glue that clots to block the fistula tract and serves as a nutrient substrate for the growth of surrounding tissue cells, leading to fistula tract coverage by fibrous scar tissue. Clinical studies have been conducted on using fibrin glue for treating low-flow tubular fistula occlusions (Lippert et al., 2011). Allogeneic-derived fibrin glue is virus-inactivated; however, it may harbor pathogens such as parvovirus B19 and Nguyen virus (Horowitz and Busch, 2008), resulting in immune responses. For example, using bovine thrombin as an activator can cause allergic reactions (Chouhan et al., 1997). Autologous-derived PRP does not contain xenogeneic or allogeneic blood-derived products; therefore, there is no risk of viral transmission or allergic reactions.

### 2.3 Surgical treatment

Since the improvement in nutritional support after the 1970s, enterocutaneous fistula surgery has changed from the first to the last choice. Typically, the fistula heals in 30% of patients with nutritional support, sepsis control, and minimal output within 4–8 weeks. A surgical plan should be initiated if healing does not occur during this period (Adaba et al., 2017). However, the process of forming a new peritoneal cavity takes approximately 6 months in a complicated abdomen. If surgery is performed before this time, the patient is at risk of further injury and complications (Grainger et al., 2018). The decision of surgical repair must be planned conscientiously, because 14%–34% of fistulas recur after surgery (Gribovskaja-Rupp et al., 2016), with a postoperative mortality rate of 10%–20% (Lynch et al., 2004). Surgical technique was an independent factor in multivariate analyses (Lynch et al., 2004). Therefore, in cases of abdominal infection, the patient’s overall condition, the type of fistula, and the possibility of self-healing should be considered when choosing the timing of surgery for an enterocutaneous fistula. Definitive surgery for enterocutaneous fistulas can only be performed if self-healing is not possible and intra-abdominal infection is controlled.

Autologous PRP containing high concentrations of platelets that is activated to release growth factors (GFs) and cytokines which have the potential capacity to modulate regenerative microenvironments, reduce inflammation and accelerate EF healing. Importantly, PRP is obtained by a centrifugation procedure to enrich platelets from autologous whole blood in a sterile environment, resulting in few complications, such as negligible immunogenicity. PRP provides a potential for repair, and has become widely used in the treatment of diseases.

## 3 PRP preparation and mechanism of action in EF treatment

PRP originated with the discovery of platelet growth factors and was developed using fibrin glue. Fibrin glue, composed of Fg, thrombin, and calcium, was first discovered in 1970 (Matras, 1970). It is a biomaterial with hemostatic and adhesive properties that can seal wounds, stop bleeding, strengthen wound contraction, and promote wound healing. Platelets contain various growth factors that promote tissue repair (Harrison and Cramer, 1993; Sánchez et al., 2003; Oyama et al., 2004; Alsousou et al., 2009; Magalon et al., 2014). Whitman et al. integrated PRP into fibrin glue and achieved good clinical results in oral and maxillofacial surgery (Whitman et al., 1997). Compared with fibrin glue, autologous PRP has many advantages, such as ease of preparation and no possibility of immune rejection and disease transmission. Furthermore, PRP contains high concentrations of growth factors and adhesion factors, such as platelet-derived growth factor (PDGF), transforming growth factor-β (TGF-β), insulin-like growth factor (IGF), epidermal growth factor (EGF), vascular endothelial cell growth factor (VEGF), Fg, fibronectin (Fn), thrombospondin (TSP) and vitronectin (Vn) (Tischler, 2002; Oyama et al., 2004; Alsousou et al., 2009), which promote tissue healing; thus, it widely used to treat clinical tissue injury or for wound healing (Alsousou et al., 2009).

### 3.1 PRP preparation

PRP preparation is based on platelet concentration. Using the different sedimentation coefficients of different blood components, PRP can be separated from whole blood by density gradient centrifugation by two methods: machine-based or manual. Machine preparation systems include the Selphyl System, Regen PRP, and GPS System (Magalon et al., 2014). The PRP obtained using different systems also differ in composition (Appel et al., 2002; Rodeo et al., 2012; Magalon et al., 2014; Degen et al., 2017; Fitzpatrick et al., 2017). Automatic equipment are professional, simple, and fast; however, their high cost limits their clinical application. Manual preparation methods include the Anitua (1999), Landesberg et al. (2000), Aghaloo et al. (2002), and Petrungaro (2001) methods, which can be divided into primary and secondary centrifugation based on the number of centrifugations. Each method involves different centrifugal forces and centrifugation times; therefore, the concentrations of platelets and growth factors in the obtained PRP also differ. Currently, secondary centrifugation is the most commonly used clinical method (Landesberg et al., 2000; Han et al., 2007; Nagata et al., 2010). The procedure of PRP preparation includes two centrifugation steps: First, the whole blood is centrifuged at a slow speed for separation into three layers, the top platelet-poor plasma layer, the middle PRP layer and the bottom red blood cell layer. Subsequently, a second centrifugation step is performed, the red blood cells are removed, the middle layer is harvested as the PRP which contains 3- to 6-fold platelet higher than the baseline of whole blood.

According to the procedures and characteristics of preparations, platelet recovery rates and platelet concentration factor changed with centrifugation force, centrifugation time and temperature, and anticoagulant use (Dhurat and Sukesh, 2014). Standardized platelet activation is a crucial step for the release of GFs and cytokines. Dugrillon et al. (2002) reported that TGF-β1 and platelet concentrations were proportionally correlated with centrifugal force when the centrifugal force was less than 800 × g. When the centrifugal force was higher than 800 × g, TGF-β1 was inversely correlated with centrifugal force. Kececi et al. (2014) suggested that individual adjustment of centrifugal force based on individual baseline values may allow for definitive platelet concentrations. Ozer et al. (2019) have shown that to obtain optimal platelet concentrations, centrifugation should be maintained for a shorter time when a higher centrifugal force is selected and for a longer time when a lower centrifugal force is selected. Temperature during processing is critical to prevent platelet activation. Macey et al. (2002) noted that cooling may retard platelet activation, and the AABB manual recommends centrifugation of blood at 21°C–24°C to obtain PRP. Most authors believe that EDTA can damage platelet membranes and recommend the use of citrate and sodium gluconate citrate as anticoagulants. Therefore, it is necessary to adopt suitable centrifugation methods and equipment (such as centrifuge tubes, reagents, and test tubes) matched to the centrifuge according to the therapeutic purpose, and to develop standardized procedures to avoid the influence of external factors on the PRP quality.

PRP dose and platelet concentrations are important matters that have to be considered in the evaluation of its biological effects. Graziani et al. (2006) compared the effects of different concentrations of PRP on fibroblast-osteoblast cell cultures and showed that the maximum effect of PRP was present at a concentration of 2.5-fold; higher concentrations of PRP resulted in decreased cell proliferation. Ra and ppl (2011) concluded that PRP concentrations up to three times the initial platelet concentration resulted in faster granulation tissue formation and better outcomes than higher concentrations, and Gentile and Garcovich (2020) recommended a platelet concentration of 1.5 × 10^6^/µL as a condition for further research *in vivo* and *in vitro*. Mazzocca et al. (2012) reported that a platelet concentration of 4–5 times the normal value can better promote bone and soft tissue repair, while the opposite effect is achieved when the concentration is too high.

PRP is widely used in dentistry, plastic surgery, orthopedics, etc. PRP injections have been used to treat musculoskeletal conditions such as rotator cuff tears, hamstring injuries, knee osteoarthritis, and Achilles tendinopathy (Santos et al., 2018). Different platelet activators can significantly influence the cytokine release kinetics in PRP. The most common method of preparing platelet gel was to add 1 ml of thrombin and 10% calcium chloride mixture(1:10) in 10 ml platelet concentration until the platelet gel was formed, which has a more sustained release pattern. Fresh platelet gel can be prepared and injected into fistula by image examination, or with assistance of endoscopy via the fistula tract.

### 3.2 The mechanism of PRP in EF treatment

**FIGURE 1:**
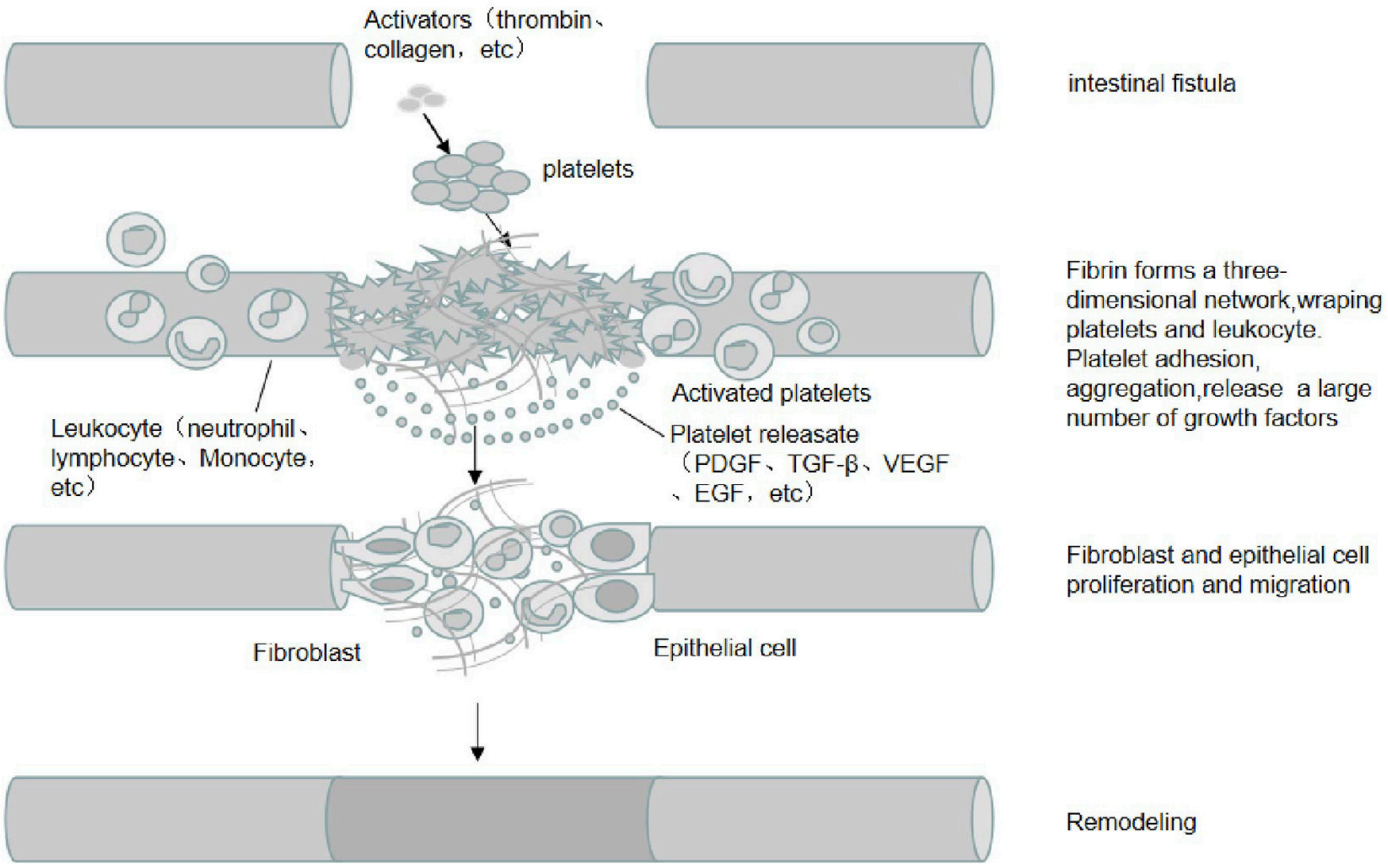
The mechanism of PRP in EF treatment. PRP is rich in high concentrations of platelets and adhesion proteins, which are activated by activators such as thrombin and collagen, in which fibrinogen is activated into fibrin and forms a three-dimensional network that wraps platelets and leukocytes. Platelets are activated and release a large number of growth factors (e.g., PDGF, TGF-β, IGF, EGF, etc.). The interaction between these growth factors and target cell surface receptors activates intracellular signaling pathways and induces the production of proteins required for regeneration, promoting the proliferation and migration of cells such as fibroblasts and epithelial cells, angiogenesis and extracellular matrix spreading, and promoting the healing of intestinal fistula tissue.

PRP has a high concentration of platelets. When activated by thrombin and collagen, platelets degranulate and release large amounts of proteins such as PDGF, TGF-β, IGF, EGF, VEGF, Fg, Fn, TSP, and Vn. These proteins include growth factors, cytokines, and chemokines. Interactions between these growth factors and surface receptors on target cells can activate intracellular signaling pathways and induce the production of proteins required for regeneration. Concurrently, these proteins can also accelerate the differentiation of mesenchymal stem cells (MSC) and promote the proliferation of osteoblasts and fibroblasts. Moreover, these proteins accelerate the synthesis of fibrin and the extracellular matrix (ECM) and promote cell chemotaxis, proliferation, differentiation, removal of tissue debris, angiogenesis, and laying of the ECM, facilitating tissue healing (Figure 1).

#### 3.2.1 The role of growth factors in PRP

PDGF, the first growth factor identified in platelets, is a cationic polypeptide with heat resistance, acid resistance, and ease of hydrolysis by trypsin. It is a specific mitogen and chemokine in cells of mesenchymal origin (fibroblasts, vascular endothelial cells, and smooth muscle cells) (Scher et al., 1979). PDGF promotes calcium ion influx, activates tyrosine kinase, adenylate cyclase, and serine/threonine kinase, increases collagenase activity, and induces gene transcription and protein expression. PDGF is a potent stimulator of vascular smooth muscle cell migration (Gerthoffer, 2007), which enhances the migration and proliferation of smooth muscle cells and endothelial cells (Ariyanti et al., 2017). In addition, PDGF promotes the proliferation and differentiation of fibroblasts during tissue remodeling (Ariyanti et al., 2017). PDGF is the earliest growth factor in wound healing. Its main functions include:1) promoting the differentiation and growth of mesenchymal cells; 2) stimulating the division of vascular endothelial cells to accelerate vascular proliferation; 3) activating collagenase to promote collagen synthesis; 4) increasing macrophages; 5) activating macrophages to promote the repair of wound sites (Antoniades, 1981).

TGF-β, composed of two polypeptide chains, acts on fibroblasts and pre-osteoblasts in the paracrine and/or autocrine form. It enhances the proliferative activity of fibroblasts, promotes the production of ECM components such as fibroblast collagen and Fn (Wrana et al., 1988; Lynch et al., 1989; Basic et al., 1996), stimulates the biosynthesis of type I collagen and Fn, and induces bone matrix deposition (Bonewald and Mundy, 1990). TGF-β can also regulate ECM synthesis and has a chemotactic effect on inflammatory cells, such as neutrophils and monocytes, to mediate local inflammatory response (Khan and Kerbel, 2018). When TGF-β acts alone or in synergy with other growth factors, endothelial cells can be induced to express α5β1 integrin, which combines with Fn to invade the ECM and promote endothelial cell migration during angiogenesis (Collo and Pepper, 1999). In addition, by affecting wound keratinocytes and epithelialization, TGF-β can stimulate fibroblast transformation into myofibroblasts and improve wound contraction and maturation (Faler et al., 2006).

VEGF is a member of the VEGF family and is an important signaling protein. VEGF signaling regulates the activity of several kinases through VEGFR1/R2 to direct cell proliferation, migration, survival, and vascular permeability during angiogenesis and induces the formation and establishment of new blood vessels for healing (Ferrara and Gerber, 2001; Apte et al., 2019). VEGF-A stimulates cell mitosis and migration. VEGF is a vasodilator that increases microvascular permeability (Ferrara and Gerber, 2001). In addition, it is important in wound repair and acts on various immune cells, including dendritic cells, T cells, regulatory T cells, and myeloid-derived suppressor cells (Khan and Kerbel, 2018), and achieves direct immunosuppressive effects on immune cells through different mechanisms.

EGF is a typical member of the peptide growth factor family and can activate EGFR by binding with a high affinity to the cell surface. EGF regulates various epithelial ion channels alone or in combination with other growth factors (Schultheiss and Diener, 2005; Trinh et al., 2007; O'Mahony et al., 2008; Zhe et al., 2011; Mroz and Keely, 2012; Girault and Brochiero, 2014). This process may trigger several biological responses, including DNA synthesis and cell proliferation, differentiation, and migration. EGF expands MSCs *in vitro* and is important in promoting paracrine signaling *in vivo* through drug-released bone marrow MSC scaffolds and repairs other tissues (Hardwicke et al., 2008; Nair et al., 2008; Tiaka et al., 2012; Arda-Pirincci and Bolkent, 2014; Rayego-Mateos et al., 2018). In addition, exogenous EGF has antiapoptotic and antioxidant effects that can alleviate tissue damage caused by ischemia/reperfusion in different organs (Hussein Ael et al., 2011; Arda-Pirincci and Bolkent, 2014; Rayego-Mateos et al., 2018).

IGF-I, also known as somatostatin-C, is a single-chain polypeptide consisting of 70 amino acids with a molecular weight of 7,650 Da (Rinderknecht and Humbel, 1978). Its structure is similar to that of proinsulin. When IGF-I binds to the IGF-1R, it activates multiple intracellular signal transduction pathways (Dupont and LeRoith, 2001; White, 2003; Vassilakos and Barton, 2018) and promotes the phosphorylation of insulin receptor substrates, thus regulating cell growth, proliferation, and metabolism. A mutual promotion exists between IGF-I and other growth factors. For example, when combined with PDGF and TGF-β, it aids DNA synthesis (Mott et al., 2002), substantially promoting osteoblast-like cell proliferation. In addition, IGF-I is a potent angiogenic factor that can induce VEGF mRNA expression in various cells (Miele et al., 2000; Akeno et al., 2002; Poulaki et al., 2003) and promote aging and cultured microvascular branches (Arthur et al., 1998). IGF-I plays multiple roles in wound healing by promoting muscle cell differentiation and angiogenesis.

#### 3.2.2 The role of adhesion proteins in PRP

In addition to platelets and leukocytes, PRP also contains abundant adhesion proteins, such as Fg, Fn, TSP, and Vn (Anitua et al., 2004; Marx, 2004), which promote the migration of osteoblasts, fibroblasts, and epithelial cells in tissue healing. Fg is a large and complex fibrous glycoprotein with three pairs of polypeptide chains, Aα, Bβ, and γ, linked to the N-terminal E domain by five symmetrical disulfide bonds (Halper and Kjaer, 2014). The fibrin peptide located in the central region is cleaved by thrombin, which converts the soluble Fg into an insoluble fibrin polymer via intermolecular interactions. These fibrins form a three-dimensional grid structure, constituting a fibrin clot to achieve hemostasis. They wrap platelets and white blood cells to prevent their loss, provide a scaffold for repairing cell crawling, and benefit growth factor secretion and tissue repair. Fg can enhance the interaction with the ECM by binding to Fn; fibrin β15-42 can mediate platelet and endothelial cell spreading and fibroblast proliferation; Fg combined with fibroblast growth factors (such as FGF-2, VEGF, interleukin IL) and cytokines can promote the proliferation of endothelial cells and enhance capillary formation (Mosesson, 2005).

Fn is an extracellular glycoprotein that exists as a soluble dimer in body fluids. Some cell-derived multimers exist in the ECM in insoluble forms (Potts and Campbell, 1994). Fn can mediate chemotaxis, cell adhesion, growth, and migration of monocytes and macrophages in peripheral blood. These functions are critical for the inflammatory, proliferative, and remodeling phases of wound repair. Additionally, Fn acts synergistically with growth factors and cytokines to enhance the effects of other growth factors on chronic wounds (Larivière et al., 2003). It combines with fibrin and rapidly accumulates at the wound site to form a temporary matrix that promotes healing during the repair process.

TSP, also known as thromboadhesin, is an ECM protein released by platelet α granules after stimulation by thrombin. It is also an “adhesion regulator” component of the ECM (Halper and Kjaer, 2014). TSPs have complex multidomain structures that can interact with different ligands, such as structural components of the ECM, cytokines, cellular receptors, growth factors, proteases, and other stromal cellular proteins. By directly or indirectly regulating the structure and activity of the abovementioned ligands, TSP achieves the corresponding responsiveness of various cells to environmental stimuli (Zhang et al., 2020), thereby participating in cell-matrix adhesion, cell migration, chemotaxis effects, inflammation, angiogenesis, and wound healing.

Vn, discovered in 1967, is an important component of human ECM and belongs to a group of adhesion glycoproteins. It is vital in cell adhesion to the surrounding matrix and regulates cell differentiation, proliferation, and morphogenesis (Preissner, 1991). In addition to its roles in cell adhesion and complement regulation, Vn exerts multiple regulatory functions in coagulation, complement, and fibrinolytic systems (Preissner and Seiffert, 1998). Vn can be recognized by cell surface receptors such as integrins, urokinase receptors, and proteoglycans, which mediate cell adhesion, migration, and invasion and are associated with tissue remodeling or bacterial tropism. When Vn interacts with the urokinase-type plasminogen activator-urokinase-type plasminogen activator receptor complex and integrin receptors, it enables smooth muscle cell migration/adhesion to the intravascular fibrous layer and increases neovascular intima formation. It is involved in old tissue (pericellular proteolysis) degradation, reorganization, and wound healing (Singh et al., 2010). Vn has the same effect on cell adhesion and bacterial binding as Fn, which can specifically bind to various bacteria and promote the adhesion of Gram-positive and Gram-negative bacteria to host cells.

The adhesion protein in PRP functions as a cell adhesion factor and cytokine. It can induce an inflammatory response in the early stages of injury, attract inflammatory cells to the wound, and exerts an inflammatory protective function. It interacts with various types of collagen, platelet growth factors, and their receptors, regulating cell migration, adhesion, aggregation, proliferation, and differentiation; participates in the repair process of various tissue injuries; and promotes EF healing.

#### 3.2.3 The role of leukocytes in PRP

Leukocytes and platelets have similar sedimentation rates during centrifugation; thus, PRP also contains high concentrations of leukocytes, such as neutrophils, monocytes, and lymphocytes (Dhurat and Sukesh, 2014; Melo et al., 2019). Neutrophils are important in the non-specific cellular immune system as the front-line body defense against the invasion of microbial pathogens. Chemokine receptors are present on neutrophil membranes. When inflammation occurs, they are attracted to the site of inflammation by chemotactic substances, followed by contact with bacteria to form phagosomes, or phagosomes containing foreign substances. Subsequently, many cytotoxic effector molecules, such as peroxide and superoxide, are produced to kill the bacteria.

Monocytes contain more non-specific lipases and, consequently, exhibit stronger phagocytosis. They eliminate pathogenic microorganisms and foreign substances that invade the body, eliminate aging and diseased cells, and regulate immune responses. Monocytes produce and release cytotoxins, interferons, and IL to participate in the body’s defense mechanisms after activation and produce factors that promote the growth of endothelial cells and smooth muscle cells. Monocytes can differentiate into macrophages with different phenotypes due to the microenvironment stimulation (Gratchev et al., 2006). M2-type macrophages produce ECM components, angiogenic factors, and chemokines involved in tissue remodeling. They can also trigger cell proliferation and repair via amine and collagen synthesis by releasing IL-10 and IL-4. In contrast, M1-type macrophages produce VEGF and fibroblasts, which exhibit bactericidal activity and inhibit cell proliferation through a nitric oxide mechanism, releasing the inflammatory cytokines IL-6 and tumor necrosis factor (TNF-α) (Ferrante and Leibovich, 2012). The topical application of PRP can promote the recruitment of macrophages from the surrounding tissues and blood, thereby promoting tissue repair (Nishio et al., 2020).

Lymphocytes are a large class of immune cells that mediate immune response. Lymphocytes elicit cellular responses to fight infections and adapt to invaders (Ubezio et al., 2014). T lymphocyte-derived cytokines (interferon-γ and IL-4) enhance macrophage polarization (Italiani and Boraschi, 2014). In combination with other leukocytes, lymphocytes promote tissue healing by regulating monocyte and macrophage differentiation (Weirather et al., 2014).

Leukocytes in PRP can help the body clear local pathogens and enhance the local anti-infection ability. They also help the body remove local necrotic tissue to speed up and promote the repair of local damaged tissue. Leukocytes secrete chemotactic growth factors from platelets, which can also secrete growth factors directly involved in tissue repair. Platelet-leukocyte interactions in PRP have anti-inflammatory tissue repair and cell signaling capabilities.

However, some researchers do not recommend leukocyte-rich platelet plasma because they believe that the concentration and composition of leukocytes in PRP affect the expression of growth factors and cytokines (Kobayashi et al., 2016). Leukocyte concentration is positively correlated with platelet derived growth factors (PDGF)-BB and VEGF concentration but is negatively correlated with FGF-b and TGF-β1 concentration. In leukocyte-rich platelet-rich plasma (LR-PRP), catabolic protease (matrix metalloproteinase-9) is highly expressed and capable of ECM remodeling. However, matrix degradation may occur when released in excess, hindering tissue healing (Zhou et al., 2015). LR-PRP has dual effects on anabolism and catabolism (Kobayashi et al., 2016). Leukocytes can increase the release of growth factors, promoting angiogenesis, matrix formation, and cell proliferation. However, abundant granulocytes and erythrocytes secrete many inflammatory mediators (such as IL-1β and TNF-α), which may have deleterious effects by increasing inflammation and delaying tissue healing (Giusti et al., 2018). Leukocytes also increase MMP levels, leading to matrix degradation. This phenomenon negatively affects the mechanical properties of the fibrin scaffolds, resulting in poorer mechanical properties and a pro-inflammatory environment. Under inflammatory conditions, the tissue promotes degradation of the fibrin network, resulting in delayed or hindered tissue repair or regeneration (Anitua et al., 2015a). Therefore, whether leukocytes have a positive or negative effect on the healing process of EF should be studied to provide sufficient evidence.

## 4 Advantages of PRP in EF treatment

The online databases PubMed and Web of Science were searched for eligible English-language studies from inception to 30 August 2023. The search strategy was conducted using Medical Subject Headings (MeSH) terms and corresponding keywords. The following search terms were used: “platelet rich plasma” [MeSH Terms] OR (“platelet rich” [All Fields] AND “plasma” [All Fields]) OR “platelet rich plasma” [All Fields] OR (“platelet” [All Fields] AND “rich” [All Fields] AND “plasma” [All Fields]) OR “platelet rich plasma” [All Fields]) AND (“intestinal fistula” [MeSH Terms] OR (“intestinal” [All Fields] AND "fistula” [All Fields]) OR “intestinal fistula” [All Fields]. We also conducted a search of the references of these articles included to identify additional studies.

Studies were eligible for inclusion if they met the following criteria: 1) the type of study was a clinical trial, case series, or retrospective study; 2) studies that enrolled patients with intestinal fistula according to clinical criteria; 3) studies that focused on the efficacy of PRP or PRP in combination with other treatments; and 4) studies that included one of the outcome metrics, such as the cure rate, the recurrence rate, the time to recovery, the number of patients with complete cure, and the adverse event Number.

The studies retrieved on PRP for the treatment of enterocutaneous fistulae were mainly case reports. (Table 1). Scala et al. (2012a) used a regenerative surgical procedure with autologous platelet gel to treat nonhealing ileocutaneous fistula, Intraoperatively, the fat tissue around the fistula and fistula tract is excised in its entirety, and platelet gel was positioned above the muscular band, and fill the superficial cavity between the fibroadipose flap and ubcutaneous tissue. The postoperative stay was only 2 days, and there were no complications, the fistula was completely repaired 6 months after surgery. In another study (Scala et al., 2012b), a vacuum-assisted closure system combined with a platelet-rich gel was used in a 75-year-old woman who had undergone a series of surgical procedures and presented with a non-healing ileo-cutaneous fistula. During the treatment, PG was applied four times approximately every 20 days. At each application, the wound area became smaller until finally the Foley catheter was removed and the remaining cavity filled with PG. The fistula was almost completely healed by the time the patient was discharged. Bai et al. (2022) used a PRP injection on a 52-year-old man who developed an enterocutaneous Fistula that did not heal for 7 months after surgery. One week after treatment, minimal secretion of the fistula was observed. The fistula closed within 2 weeks following treatment with PRP. At 6-month follow-up, the patient no longer felt abdominal distension and abdominal pain, and no complications had been reported.

**TABLE 1:** Basic information of included studies.

Author	Year	Research design	Therapeutic method	Type of fistula	Number of treatments	Cured or not	Complication or not
Scala et al.	2012	Case Report	PG	ileocutaneous fistula	1	Cured	not
Scala et al.	2012	Case Report	PG + VAC	ileocutaneous fistula	4 times/20 days	completely healed	No description
Bai et al.	2022	Case Report	PRP	enterocutaneous fistula	1	Cured	not

The retrieved cases have shown that PRP provides a potential for repair, and have the potential capacity to improve tissue anabolism for regeneration which are key in EF healing. Several PRP studies have reported promising results in the treatment of many fistulas. Damien et al. (2022) treated two patients with persistent tracheoesophageal fistulae after total laryngectomy with local injections of autologous PRP, and both observed complete closure of the fistula. No treatment-related side effects were observed. Wu et al. (2021) treated three patients with tracheobronchial fistulas with fistula diameters of 4–8 mm with autologous PRP, all of whom were successfully healed without detectable treatment-related complications or fistula-related symptoms. In a prospective study, Wu et al. (2014) grouped 145 patients with tubular EF of various causes to evaluate the efficacy and safety of autologous platelet-rich Fg glue for blocking EF. Hermann et al. (2022a) treated 13 patients with ulcerative colitis rectovaginal fistulae using PRP between 2018 and 2020, and nine (69%) patients achieved complete closure of the RVF. The fistula remained closed for 6–12 months. Other studies have confirmed the effectiveness of PRP in the treatment of anal and rectovaginal fistulas (Hermann et al., 2022b; Cwaliński et al., 2023). PRP is rich in platelets and releases many cytokines, growth factors, and microparticle exosomes after activation. These factors exert paracrine effects on different cell types, promoting cell proliferation, stimulating angiogenesis and cell migration, and promoting tissue regeneration. PRP contains much Fg, which forms a three-dimensional, biocompatible fibrin scaffold after activation. This fibrin scaffold wraps platelets and white blood cells to prevent their loss and provides a biological scaffold for migrating fibroblasts and endothelial cells to accelerate cell integration. Platelets also secrete antimicrobial peptides, which have antibacterial and anti-inflammatory effects. The effects of PRP on tissue healing, its inhibition of the inflammatory response, resistance to bacterial effects, and good histocompatibility provide a theoretical foundation for PRP as an ideal material for tubular EF closure.

The advantages of PRP in EF treatment can be summarized as follows: 1) PRP is an autologous platelet concentrate that releases many growth factors after activation. These growth factors are close to physiological conditions and can synergistically promote tissue repair; 2) PRP can continuously release growth factors and secrete more than 95% of presynthetic growth factors within 1 h after activation. The platelets continue to synthesize and secrete more cytokines and growth factors from their mRNA reserves over 7 days (Marx, 2004), thereby promoting EF healing; 3) PRP contains a large amount of Fg, which forms a three-dimensional network structure after activation. This structure provides a good scaffold for repairing cells (Mosesson, 2005) and limits growth factors to the fistula, which benefits EF healing; 4) PRP is an autologous preparation that avoids immune rejection, disease transmission, and allergic reactions caused by exogenous growth factors (Marx, 2004); 5) The sedimentation coefficients of leukocytes and platelets in the blood are similar; therefore, PRP produced by centrifugation contains many leukocytes. The pH of PRP is 6.5 to 6.7 (Marx, 2004), which is acidic. Platelets also contain different bacteriostatic proteins that inhibit bacterial growth and prevent infection (Yeaman, 1997; Tang et al., 2002); 6) PRP preparation is simple, less traumatic, inexpensive, and can effectively reduce medical expenses; 7) PRP has good biocompatibility, biodegradability, and degradability in the body.

## 5 Influencing factors of PRP in EF treatment

The main factors determining the effect of PRP on EF are the number and function of platelets. Therefore, all factors affecting platelets’ number and function may affect efficacy. Individual differences, such as patient age, sex, basal platelet count and function, disease state, different PRP preparation methods, and different activation methods, result in differences in the concentrations of various PRP components and may affect the therapeutic effect. Nonsteroidal anti-inflammatory or antiplatelet drugs can inhibit platelet function and theoretically inhibit PRP effect. However, no clear evidence has been provided in clinical studies. In a study of 12 healthy male participants, aspirin inhibited the AA-mediated release of VEGF, TGF-β1, and PDGF-AB in PRP after 2 weeks of low-dose daily aspirin (81 mg). However, it did not affect the thrombin-mediated release of VEGF and TGF-β1, and only partial inhibition of PDGF-AB was observed (Jayaram et al., 2019). Another study revealed that PDGF-AA, PDGF-AB, and IL-6 expression were considerably reduced in the PRP of patients taking naproxen. In contrast, the expression of TNF-α, IL-1b, IL-8, VEGF, and dFGF-2 was not substantially decreased. PDGF-AA, PDGF-AB, and IL-6 returned to baseline levels 1 week after discontinuation (Mannava et al., 2019), suggesting that discontinuing nonsteroidal anti-inflammatory drugs for at least 1 week before LR-PRP therapy may improve the levels of some biological factors. Some studies have reported no obvious effects of PRP when using anti-inflammatory or antiplatelet drugs. For example, some researchers investigated patients undergoing cardiac surgery who received aspirin, aspirin plus clopidogrel, or no antiplatelet therapy. Compared with the control group, the levels of PDGF-BB and TGF-β1 in PRP of patients in both experimental groups were not significantly increased (Smith et al., 2007). Another group of researchers discovered no obvious difference between the experimental and control groups for patients treated with acetylsalicylic acid, acamarool, glucosamine sulfate, or glucosamine sulfate plus chondroitin sulfate and isolated PRGF, and platelet activation in PRGF and amounts of PDGF-AB, TGF-β1, VEGF and hepatocyte growth factor. In addition, no difference was observed in cell migration or proliferation between the experimental and control fibroblasts after treatment with PRGF (Anitua et al., 2015b). The complex biological processes of platelets make it difficult to obtain clear conclusions regarding the effect of antiplatelet therapy on the biological activity and efficacy of PRP. Thus, more efforts should be devoted to this area. PRP preparation requires a sterile operating environment. During the preparation process, blood can be exposed to the external environment during transfer. If the environment is not completely disinfected, blood contamination may occur, affecting the therapeutic effect.

## 6 Conclusion and outlook

In conclusion, we have reviewed the classification and treatment of EF, and PRP preparation and mechanism of action in EF treatment. PRP therapy has been shown to enhance tissue regeneration and promote wound healing, which makes it a potential therapeutic option for EF. Although PRP has achieved certain therapeutic effects in the treatment of EF, there are still many problems to be addressed. For example, there is a significant disparity in the preparation standards, injection dosage, and evaluation indicators of PRP, and there is no consistent standard. Standardized preparation protocols for PRP are crucial for reproducing consistent and accurate results in future research. Additionally, there is currently no literature describing the half-life of PRP’s therapeutic effect on EF. Some patients in clinical settings may require a second PRP treatment if the first one did not yield satisfactory results, and the impact of the time interval between the two treatments on EF repair remains to be clarified. Therefore, studying the concentration gradient of PRP, the number of treatments, and the time interval between treatments can help improve the clinical application of PRP in EF treatment. Currently, most clinical studies have small sample sizes, some control variables are not controlled, and the follow-up time is short. As a result, there is a lack of strong evidence for the effectiveness, safety, and adverse effects of PRP in the treatment of EF, ultimately leading to a low level of evidence in clinical research. Therefore, high-quality, well-designed randomized controlled trials and *in vitro* experiments are still needed to further supplement and improve the quality of evidence for the clinical application of PRP in the treatment of EF.
